# One-Week Elderberry Juice Intervention Promotes Metabolic Flexibility in the Transcriptome of Overweight Adults During a Meal Challenge

**DOI:** 10.3390/nu17193142

**Published:** 2025-10-01

**Authors:** Christy Teets, Andrea J. Etter, Patrick M. Solverson

**Affiliations:** 1 Department of Nutrition and Exercise Physiology, Elson S. Floyd College of Medicine, Washington State University, Spokane, WA 99202, USA; christy.teets@wsu.edu; 2 Department of Nutrition and Food Science, University of Vermont, Burlington, VT 05405, USA; andrea.etter@uvm.edu

**Keywords:** elderberry juice, transcriptome, metabolic flexibility, berry anthocyanins, functional foods, meal tolerance test, obesity, PBMC

## Abstract

Background: Metabolic flexibility, the ability to efficiently switch between fuel sources in response to changing nutrient availability and energy demands, is recognized as a key determinant of metabolic health. In a recent randomized controlled human feeding trial, overweight individuals receiving American black elderberry juice (EBJ) demonstrated improvements in multiple clinical indices of metabolic flexibility, but the mechanisms of action were unexplored. The objective of this study was to utilize RNA sequencing to examine how EBJ modulates the transcriptional response to fasting and feeding, focusing on pathways related to metabolic flexibility. Methods: Overweight or obese adults (BMI > 25 kg/m^2^) without chronic illnesses were randomized to a 5-week crossover study protocol with two 1-week periods of twice-daily EBJ or placebo (PL) separated by a washout period. RNA sequencing was performed on peripheral blood mononuclear cells from 10 participants to assess transcriptomic responses collected at fasting (pre-meal) and postprandial (120 min post-meal) states during a meal-challenge test. Results: The fasted-to-fed transition for EBJ showed 234 differentially expressed genes following EBJ consumption compared to 59 genes following PL, with 44 genes shared between interventions. EBJ supplementation showed significantly higher enrichment of several metabolic pathways including insulin, FoxO, and PI3K–Akt signaling. KEGG pathway analysis showed 27 significant pathways related to metabolic flexibility compared to 7 for PL. Conclusions: Our findings indicate that short-term elderberry juice consumption may promote metabolic flexibility in overweight adults.

## 1. Introduction

Metabolic flexibility, the ability to efficiently switch between fuel sources in response to changing nutrient availability and energy demands, is recognized as one key determinant of metabolic health. This adaptive capacity enables healthy individuals to preferentially utilize glucose in the fed state and shift toward fatty acid oxidation during fasting. The maintenance of this flexibility is fundamental, as it supports optimal energy balance and cellular resilience across physiological conditions [1,2,3,4].

Loss of metabolic flexibility is a driver of metabolic disorders. Metabolic inflexibility precedes and contributes to the development of chronic conditions such as type 2 diabetes, obesity, hepatic steatosis, and cardiovascular disease [3,4]. Rather than viewing insulin resistance and related comorbidities as triggers of inflexibility, empirical evidence emphasizes that the impaired substrate switching itself initiates a cascade of complications—disrupting insulin action, energy homeostasis, mitochondrial function, and inflammatory control [3,5]. This paradigm shift places the restoration of metabolic flexibility at the center of preventive and therapeutic strategies for metabolic disease [2].

Anthocyanins, the predominant polyphenolic pigments found in berries, are increasingly studied for their role in improving metabolic flexibility. Under certain conditions, anthocyanins modulate insulin signaling, mitochondrial function, inflammation, and nutrient-sensing pathways (e.g., AMPK, PPARs), which restore or enhance fuel switching in obesity or insulin-resistant states [6,7,8].

American black elderberries (*Sambucus nigra* spp. *canadensis*) have the highest anthocyanin concentrations compared to commonly consumed fruits and exhibit the ability to improve metabolic health in some studies [9]. In our recent randomized controlled human feeding trial involving overweight adults [10], individuals receiving American black elderberry juice (EBJ) demonstrated improvements in multiple indices of metabolic flexibility. These improvements were marked by reduced postprandial glucose and insulin levels, and a decline in respiratory quotient (RQ) in the fasted-to-fed transition, relative to placebo (PL). This suggested enhanced metabolic flexibility without changes in caloric intake or body weight. These findings indicated that EBJ may support dynamic fuel switching while enhancing physiological responses critical for overall well-being. Yet, the underlying molecular mechanisms that drive the effect remain unclear.

Building upon our prior work, our study objective is to investigate these molecular mechanisms by analyzing gene expression profiles from peripheral blood mononuclear cells (PBMCs) collected from participants during a meal tolerance test from the previous clinical study. Using RNA sequencing, we examined how EBJ modulates the transcriptional response to fasting and feeding, focusing particularly on pathways related to metabolic flexibility.

## 2. Materials and Methods

### 2.1. Human Clinical Trial

Details of the human clinical trial (NCT 06626373) were previously published [10]. Briefly, 18 overweight and obese adults (15 female) completed a 7-day randomized, diet- and placebo-controlled, crossover trial comparing twice-daily consumption of 355 g of 100% EBJ containing 720 mg/day cyanidin-3-glucoside equivalents (C3GE) or a matched placebo beverage. Each arm included four days of a 100% fully investigator-controlled diet, followed by a three-week washout before crossover. Participants maintained habitual physical activity, completed daily compliance questionnaires, and returned empty food and beverage containers. A 3 h meal tolerance test (MTT) was administered on the morning of day 8 of each arm. The MTT included a high-carbohydrate breakfast (~60 g sugar from waffles and syrup) plus a full daily dose of either EBJ or PL. Serum was collected every 30 min for 3 h. The study was approved by the Washington State University IRB (WSU-IRB 18597) and conducted in accordance with the Declaration of Helsinki, and written informed consent was obtained from all participants.

### 2.2. RNA Sequencing

RNA sequencing was performed on PBMCs from 10 participants to assess transcriptomic responses. Fasting (pre-meal) and postprandial (120 min post-meal) samples were collected into PAXgene Blood RNA Tubes (Qiagen, Valencia, CA, USA), yielding 40 libraries across fasted and fed states in both treatment arms. RNA was extracted using the PAXgene Blood RNA Kit (Qiagen, Hilden, Germany). Quality control and sequencing were completed by Novogene (Sacramento, CA, USA). All samples had RNA integrity numbers (RIN) above 7.8 (mean = 8.6). Globin mRNA was depleted using the QIAseq FastSelect-Globin Kit, followed by poly (A) selection. Libraries were prepared with the NEBNext Ultra II Directional RNA Library Prep Kit (New England Biolabs, Ipswich, MA, USA) and sequenced on an Illumina NovaSeq 6000 S1 platform (2 × 150 bp paired-end reads), targeting approximately 20 million read pairs per sample.

### 2.3. Read Processing and Alignment

RNA-seq data were processed using Galaxy (v23.1.5dev0) [11]. Quality assessment was performed with FastQC to check read integrity, GC content, and adapter contamination. Trimmomatic removed low-quality bases, adapters, and short or degraded reads [12]. Ribosomal RNA was filtered using SortMeRNA (v2.1), removing 2–5% of reads per sample to focus on mRNA [13]. Cleaned reads were aligned to the GRCh38 human genome using HISAT2 [14], with alignment rates between 87.9% and 98.1%, indicating high mapping quality. Gene-level quantification was performed using featureCounts (v2.0.1) [15] with paired-end, strand-specific settings and Ensembl GRCh38.113 annotations. Multimapping and ambiguous reads were excluded for specificity.

### 2.4. Differential Gene Expression Analysis 

Differential gene expression was analyzed in R (v4.4.1) using DESeq2 and lmerSeq. Genes with fewer than 10 reads in at least four samples were excluded. The variance-stabilizing transformation (VST) was applied with DESeq2 [16] and Ensembl gene IDs were mapped to gene symbols using org.Hs.eg.db [17]. Due to the 2 × 2 crossover design and repeated sampling, linear mixed-effects models were fit using lmerSeq, with the following model:

~Treatment * State + Sequence + (1 | Participant)

where Treatment refers to EBJ vs. PL and State refers to fasted vs. fed. To evaluate potential carryover effects inherent to 2 × 2 crossovers, we included Sequence (EBJ → PL vs. PL → EBJ) as a fixed effect, consistent with recommended practice for crossover designs [18]. Sequence tests were adjusted using Benjamini–Hochberg procedures. A random intercept for Participant modeled within-subject correlations. This model aligns with recent recommendations emphasizing the importance of modeling interaction effects and repeated measures in RNA-seq studies [19,20]. Because contrasts are on the VST scale, effect estimates approximate log2 fold-changes. Genes with BH-adjusted *p* < 0.05 and |log2FC| > 0.25 (~19%) were considered differentially expressed. This aims to balance sensitivity and error control for PBMC datasets, where nutritionally induced responses are often present as small per-gene shifts [21,22]. The analysis included the main effects of treatment and metabolic state, their interaction, and pairwise contrasts (e.g., EBJ vs. PL in the fed state). This model-based approach enabled robust detection of expression changes while accounting for inter-individual variability and study design.

### 2.5. Pathway-Based Filtering of Differentially Expressed Genes

To identify gene expression changes relevant to metabolic regulation, we applied a curated, pathway-driven filtering strategy based on Kyoto Encyclopedia of Genes and Genomes (KEGG) and literature-defined metabolic functions. First, differentially expressed genes (DEGs) with adjusted *p*-value (padj) <0.05 and absolute log_2_ fold-change > 0.25 were retained. A custom metabolic flexibility gene whitelist was constructed by aggregating all human genes from 38 KEGG pathways associated with energy metabolism, nutrient sensing, redox regulation, and mitochondrial function (e.g., glycolysis, TCA cycle, insulin signaling, AMPK, autophagy) (Supplementary Table S1). Pathways were queried via KEGG’s REST API, and gene symbols, descriptions, and annotations were extracted.

In addition, 74 literature-curated regulators of metabolic flexibility—representing core nodes in pathways such as glucose uptake, fatty acid oxidation, mitochondrial biogenesis, and immunometabolism—were manually added to ensure inclusion of biologically important genes not annotated in KEGG [23,24,25]. These genes were cross-referenced against the KEGG-derived whitelist, and only those not already represented were retained to avoid duplication. Genes were then filtered by presence in this expanded, non-redundant whitelist.

Filtered DEGs from each condition (EBJ and PL) were annotated with pathway information derived from the whitelist and grouped by KEGG pathway category or literature-defined function. For each pathway, we computed the number of DEGs detected per group and quantified the overlap in gene membership between conditions. To assess whether the degree of gene sharing between groups was greater than expected by chance, we used Fisher’s exact test on a 2 × 2 contingency table of shared vs. unique genes.

### 2.6. Gene Ontology Enrichment Analysis

Gene Ontology (GO) enrichment analysis was conducted using the enrichGO function from the clusterProfiler package [26] with the org.Hs.eg.db annotation database [17]. DEGs were filtered at an adjusted *p*-value threshold of < 0.05 and converted from gene symbols to Entrez IDs using bitr (). GO enrichment was run across the Biological Process (BP), Molecular Function (MF), and Cellular Component (CC) ontologies using a contrast-specific background gene list derived from each raw count matrix. Enrichment parameters included a Benjamini–Hochberg adjusted *p*-value cutoff of 0.05.

To assign directionality to enriched GO terms, contributing gene symbols were decomposed and cross-referenced with DEG direction. Each GO term was annotated as “upregulated”, “downregulated”, or “mixed” based on the majority direction of its associated genes. The top 10 terms per contrast and ontology were visualized based on −log_10_(FDR). Cluster-level keywords were automatically extracted from GO term descriptions to summarize biological themes within clusters.

### 2.7. Gene Set Enrichment Analysis

Gene set enrichment analysis (GSEA) was performed to identify pathways relevant to metabolic flexibility that were altered by EBJ and PL treatments across nutritional states. Genes were ranked by either estimated log_2_ fold-change or t-statistics, depending on the contrast, and mapped to Entrez Gene IDs using the bitr () function from the clusterProfiler package with the org.Hs.eg.db annotation database. Enrichment was conducted using the gseKEGG () function, focusing on curated KEGG pathways. Because GSEA is a rank-based method, it considers the distribution of all expressed genes rather than relying on a threshold-defined DEG list. This approach reduces the dependency of pathway enrichment results on absolute DEG counts and enables more robust comparisons across conditions with differing DEG numbers.

To prioritize biologically relevant results, GSEA outputs were filtered using a manually curated whitelist of metabolism- and signaling-related KEGG pathways (*n* = 51), including canonical pathways (e.g., glycolysis, insulin signaling, AMPK, TCA cycle) as well as additional terms identified through enrichment results and manual review (e.g., pantothenate and CoA biosynthesis, sphingolipid metabolism, oxidative phosphorylation).

Results were further filtered by a significance threshold of FDR-adjusted *p*-value < 0.1, and pathway directionality was recorded using normalized enrichment scores (NES).

## 3. Results

### 3.1. DEG Main Effects and Contrasts

**Figure 1:**
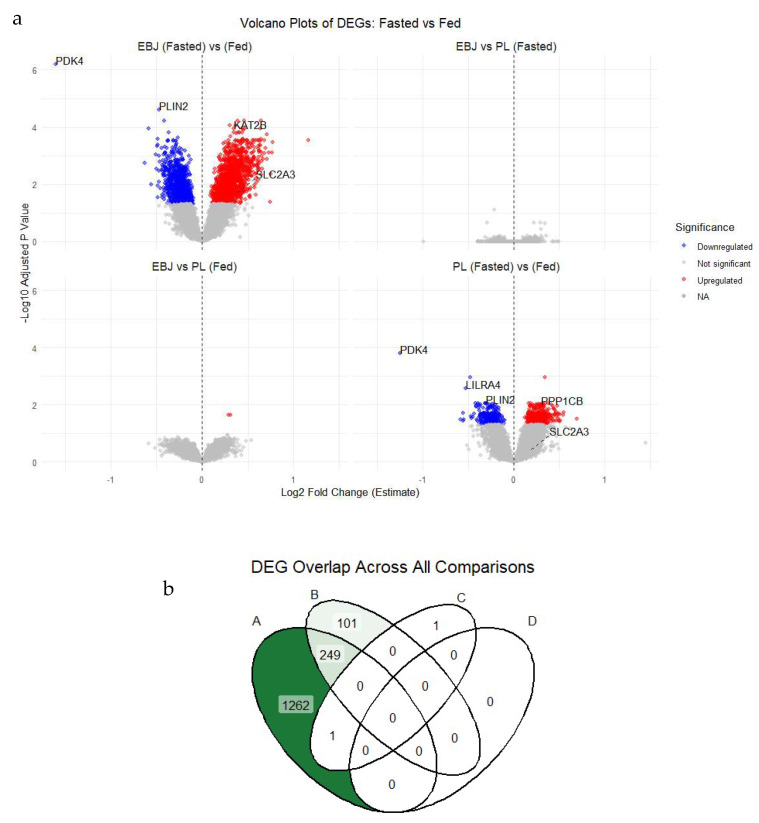
Differentially expressed genes (DEGs) across treatment contrasts. (**a**): Volcano plots of DEGs identified in each of the four contrasts. The x-axis shows the log_2_ fold change (log_2_FC) in gene expression, and the y-axis shows the −log_10_ adjusted *p*-value (false discovery rate, FDR). Each point represents a gene: red = significantly upregulated, blue = significantly downregulated, grey = not significant. DEGs were defined as those with FDR-adjusted *p* < 0.05 and |log_2_FC| > 0.25. Gene labels indicate selected DEGs of interest. (**b**): Venn diagram showing overlap of significant DEGs across the four contrasts: (A) EBJ fasted → fed transition, (B) placebo (PL) fasted → fed transition, (C) EBJ vs. PL under fed conditions, and (D) EBJ vs. PL under fasted conditions. Overlapping regions indicate genes shared between contrasts, with counts shaded from white to dark green proportional to the size of the overlap. Data reflect pooled transcriptomic analyses from *n* = 10 participants per group.

The transcriptomic analysis results are provided in Supplementary Table S2 and visualized in Figure 1. During the fasted-to-fed transition, a total of 1512 genes were identified as differentially expressed (FDR-adjusted *p* < 0.05, |log_2_FC| > 0.25) in the EBJ group, while 350 DEGs were identified in the PL group. Direct comparisons between EBJ and PL in the fed state yielded two DEGs, while no significant DEGs were identified in the fasted state or for the treatment × state interaction term.

In Figure 1a, the distribution of fold changes for EBJ fasted vs. fed comparisons highlights changes ranging from −2.6 to +2.8, with 410 genes exhibiting a log_2_FC greater than ±0.32, equating to at least a 1.25-fold change. The overlap of DEGs EBJ and PL feeding responses is shown in Figure 1b, with 249 DEGs shared and 1262 unique to the EBJ group. These findings indicate that while baseline gene expression levels in the fasted state were comparable between EBJ and PL, the EBJ group demonstrated approximately four times more DEGs activated in response to feeding than the PL group.

### 3.2. Metabolic Flexibility Gene Expression

To examine pathway-level shifts in metabolic gene regulation during the fasted-to-fed transition, differentially expressed genes (DEGs; FDR < 0.05) were mapped to KEGG and curated literature-defined pathways. This analysis yielded 234 metabolic DEGs in the EBJ group and 59 in the PL group, with 44 DEGs shared between groups (Supplementary Table S3). No significant metabolic DEGs were identified in the direct EBJ vs. PL contrast.

To compare the functional distribution of DEGs, Fisher’s exact test was applied to identify pathways with significant differences in gene representation between treatment arms (Table 1). Several pathways showed statistically significant enrichment in both groups, but with greater gene-level representation in the EBJ group. This included insulin signaling (FDR = 1.6 × 10^−5^), FoxO signaling (FDR = 0.0004), MAPK signaling (FDR = 0.0012), circadian rhythm (FDR = 0.0015), PI3K–Akt signaling (FDR = 0.0090), and autophagy (FDR = 0.0135). Pathways related to cytokine–cytokine receptor interaction (FDR = 0.0153), fatty acid degradation (FDR = 0.0255), and fatty acid biosynthesis (FDR = 0.0270) also showed significantly higher overlap in EBJ-fed samples (Supplementary Table S4 full Fisher’s results).

Among the top five significantly regulated genes in both treatment groups (FDR ≤ 0.05), three genes were shared: PDK4, PLIN2, and LILRA4, each showing stronger regulation in the EBJ group. PDK4, which encodes pyruvate dehydrogenase kinase 4, was more strongly downregulated in EBJ-fed samples (estimate = −1.61, FDR = 6.3 × 10^−7^) compared to PL-fed samples (estimate = −1.25, FDR = 1.6 × 10^−4^). PLIN2 (perilipin 2), involved in lipid droplet formation and lipid mobilization, was downregulated by −0.47 (FDR = 2.5 × 10^−5^) in EBJ and −0.31 (FDR = 0.0091) in PL. Similarly, LILRA4, which encodes leukocyte immunoglobulin-like receptor A4 and is involved in immune cell signaling, showed greater downregulation in EBJ (estimate = −0.59, FDR = 1.1 × 10^−4^) than in PL (estimate = −0.53, FDR = 0.0027).

In addition to these shared genes, EBJ-fed samples uniquely upregulated SLC2A3 (estimate = 0.39, FDR = 0.019), a gene that encodes the glucose transporter protein GLUT3, which is responsible for transporting glucose across cell membranes, and PPARGC1B (estimate = 0.64, FDR = 1.1 × 10^−4^), a coactivator of genes involved in mitochondrial biogenesis and energy metabolism. In contrast, the PL group showed unique regulation of ACAA2 (estimate = −0.32, FDR = 0.0091), which encodes acetyl-CoA acyltransferase 2 and is involved in mitochondrial fatty acid β-oxidation, and PPP1CB (estimate = 0.30, FDR = 0.0098), a catalytic subunit of protein phosphatase 1 that contributes to cell cycle regulation, insulin signaling, and circadian processes.

### 3.3. Gene Ontology Enrichment Analysis

**Figure 2:**
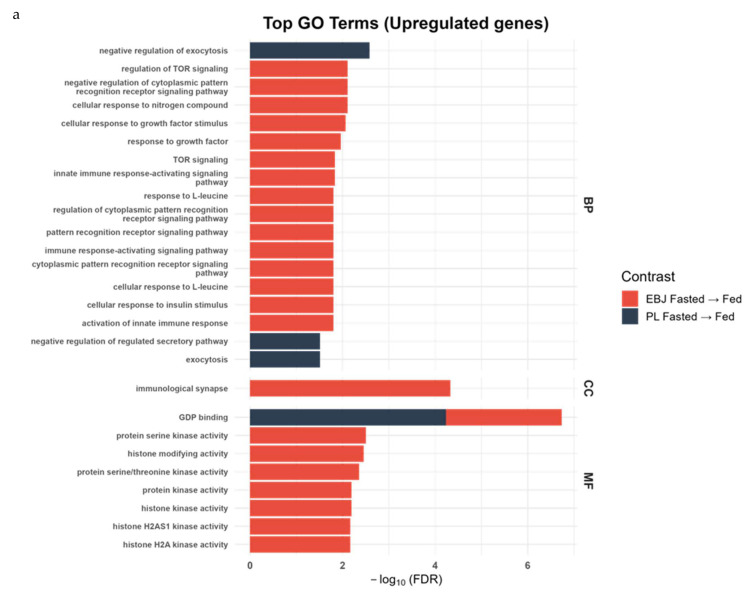

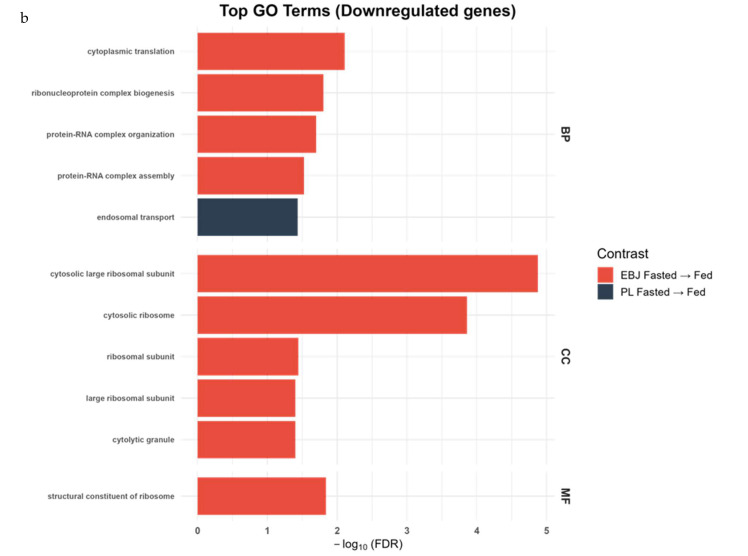
Gene Ontology enrichment for differentially expressed genes (DEGs) in response to feeding. (**a**). Bar plot showing the top Gene Ontology (GO) terms enriched among upregulated DEGs during the fasted-to-fed transition. GO categories are Biological Process (BP), Cellular Component (CC), and Molecular Function (MF). Results are stratified by treatment group (red: elderberry juice, EBJ; black: placebo, PL). Bars represent the –log_10_ of the false discovery rate (FDR)-adjusted *p*-value for each enriched term. (**b**). GO enrichment for downregulated DEGs in response to feeding, in the same format as Figure (**a**). Data reflect pooled transcriptomic analyses from *n* = 10 participants per group.

Gene ontology (GO) enrichment analysis was conducted to identify coordinated cellular responses to the fasted-to-fed transition in each treatment group, revealing functional trends in DEGs (Figure 2a,b; Supplementary Table S5).

In the EBJ group, numerous GO biological process (BP) terms were significantly enriched among upregulated genes (FDR < 0.05), including pathways related to immune signaling, hormone response, and nutrient sensing. Specifically, significant enrichment was observed in cellular response to nitrogen compound (GO:1901699, FDR = 0.0073, n = 133), regulation of TOR signaling (GO:0032006, FDR = 0.0073, n = 48), response to growth factor (GO:0070848, FDR = 0.0102, n = 136), cellular response to insulin stimulus (GO:0032869, FDR = 0.0147, n = 56), and insulin receptor signaling pathway (GO:0008286, FDR = 0.0451, n = 37). Immune-related pathways such as innate immune response-activating signaling pathway (GO:0002758, FDR = 0.0138, n = 78) and pattern recognition receptor signaling pathway (GO:0002221, FDR = 0.0147, n = 73) were also significantly upregulated. In contrast, downregulated processes included ribonucleoprotein complex biogenesis (GO:0022613, FDR = 0.0147, n = 121), protein–RNA complex organization (GO:0071826, FDR = 0.0187, n = 60), and protein–RNA complex assembly (GO:0022618, FDR = 0.0280, n = 57).

For the Cellular Component (CC) category, downregulated genes in the EBJ group were significantly enriched in multiple ribosome-related compartments, including the cytosolic large ribosomal subunit (GO:0022625, FDR = 1.25 × 10^−5^, n = 29), cytosolic ribosome (GO:0022626, FDR = 1.29 × 10^−4^, n = 44), ribosomal subunit (GO:0044391, FDR = 3.37 × 10^−2^, n = 55), large ribosomal subunit (GO:0015934, FDR = 3.72 × 10^−2^, n = 37), and cytolytic granule (GO:0044194, FDR = 3.72 × 10^−2^, n = 9). The immunological synapse (GO:0001772, FDR = 4.36 × 10^−5^, n = 25) was significantly enriched among upregulated genes.

Within the Molecular Function (MF) category, upregulated genes in the EBJ group were significantly enriched for several kinase-related activities, including protein serine/threonine kinase activity (GO:0004674, FDR = 4.19 × 10^−3^, n = 104), protein kinase activity (GO:0004672, FDR = 6.08 × 10^−3^, n = 127), histone kinase activity (GO:0035173, FDR = 6.09 × 10^−3^, n = 68), and histone H2A kinase activity (GO:0140995, FDR = 6.45 × 10^−3^, n = 65). Additional significant enrichment was observed for GDP binding (GO:0019003, FDR = 2.98 × 10^−3^, n = 27) and histone-modifying activity (GO:0140993, FDR = 3.30 × 10^−3^, n = 109). In contrast, the structural constituent of the ribosome (GO:0003735, FDR = 1.37 × 10^−2^, n = 50) was enriched among downregulated genes.

In the placebo group, GO enrichment analysis indicated significant changes in the BP categories in response to feeding. Upregulated BP included negative regulation of exocytosis (GO:0045920, FDR = 0.0025, n = 10) and negative regulation of the regulated secretory pathway (GO:1903306, FDR = 0.0299, n = 7), both of which included RAP and RAB GTPase genes. Positive regulation of neuron projection development (GO:0010976, FDR = 0.0299, n = 17) was also enriched, though regulation within this term was mixed. Broader secretory pathway terms, such as exocytosis (GO:0006887, FDR = 0.0299, n = 29) and endosomal transport (GO:0016197, FDR = 0.0359, n = 28), were enriched, with the latter primarily associated with downregulated genes.

Within the MF category for the placebo group, GDP binding (GO:0019003, FDR = 5.85 × 10^−5^, n = 15) was significantly enriched among upregulated genes, encompassing multiple small GTPases (RAB8B, RAP1A, RAP1B, RHEB), suggesting alterations in vesicular trafficking activity during the fed state.

PL did not show any significant changes in the CC category.

### 3.4. Gene Set Enrichment Analysis

To explore coordinated transcriptional responses to feeding, we performed KEGG GSEA on ranked gene expression data from fasted and fed samples in both the EBJ and PL arms (Supplementary Table S6). Enrichment was performed using the gseKEGG () function, and results were filtered using a manually curated whitelist of 51 KEGG pathways relevant to metabolic flexibility. Pathways of interest included canonical metabolic and signaling routes (e.g., insulin signaling, oxidative phosphorylation, AMPK, TCA cycle) and were retained if the FDR-adjusted *p*-value was below 0.1 (Supplementary Table S7).

**Figure 3:**
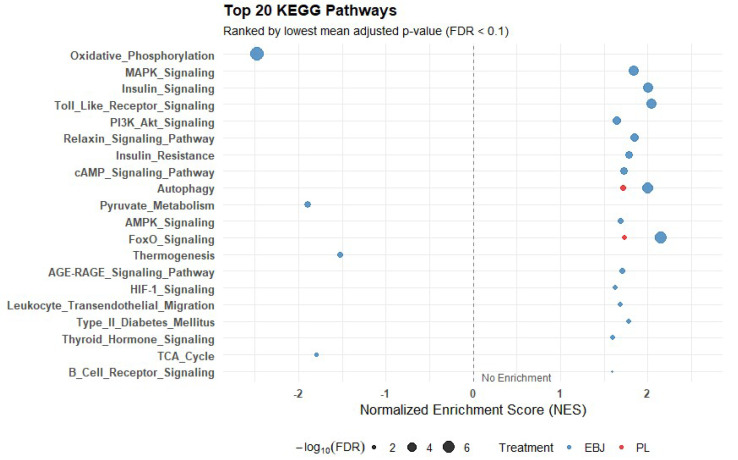
Top KEGG pathways identified by gene set enrichment analysis. Gene set enrichment analysis (GSEA) of the top 20 Kyoto Encyclopedia of Genes and Genomes (KEGG) pathways in peripheral blood mononuclear cells (PBMCs) following elderberry juice (EBJ) or placebo (PL) treatment. Pathways are ranked by the lowest mean adjusted *p*-value across both groups [false discovery rate (FDR) < 0.1]. GSEA evaluates the distribution of all expressed genes ranked by fold-change or test statistic. Each dot represents a pathway–treatment combination, with the *x*-axis showing the normalized enrichment score (NES), a standardized metric reflecting the degree to which a pathway’s genes are overrepresented at the top or bottom of the ranked list. Dot size corresponds to statistical significance [−log_10_(FDR)], and color indicates treatment group (blue = EBJ, red = PL). Positive NES values indicate pathway enrichment in the fasted versus fed state, while negative NES values indicate relative downregulation. The vertical dashed line at NES = 0 denotes no enrichment. Data reflect pooled transcriptomic analyses from *n* = 10 participants per group.

In the EBJ group, 27 pathways met the significance threshold (FDR < 0.1), of which 24 had FDR < 0.05. Figure 3 illustrates a dotplot of the top 20 pathways. Upregulated pathways included FoxO signaling (NES = 2.15, FDR = 8.67 × 10^−7^), insulin signaling (NES = 2.00, FDR = 3.79 × 10^−5^), PI3K–Akt signaling (NES = 1.65, FDR = 0.0003), mTOR signaling (NES = 1.76, FDR = 0.0012), AMPK signaling (NES = 1.68, FDR = 0.0037), cAMP signaling (NES = 1.72, FDR = 0.0013), and cGMP-PKG signaling (NES = 1.82, FDR = 0.0003). Additional significantly upregulated pathways included autophagy (NES = 1.99, FDR = 4.20 × 10^−6^), Toll-like receptor signaling (NES = 2.05, FDR = 4.82 × 10^−5^), HIF-1 signaling (NES = 1.63, FDR = 0.0064), and adipocytokine signaling (NES = 1.64, FDR = 0.020).

Downregulated pathways in the EBJ group included oxidative phosphorylation (NES = −2.47, FDR = 1.16 × 10^−8^), TCA cycle (NES = −1.79, FDR = 0.011), pyruvate metabolism (NES = −1.89, FDR = 0.0031), fatty acid degradation (NES = −1.55, FDR = 0.041), and glycolysis/gluconeogenesis (NES = −1.47, FDR = 0.058).

In the PL group, seven pathways passed the FDR < 0.1 threshold. These included upregulation of FoxO signaling (NES = 1.74, FDR = 0.0075), autophagy (NES = 1.72, FDR = 0.0056), mTOR signaling (NES = 1.45, FDR = 0.068), and cGMP-PKG signaling (NES = 1.56, FDR = 0.039). Downregulated pathways included glycolysis/gluconeogenesis (NES = −1.52, FDR = 0.097) and fructose and mannose metabolism (NES = −1.64, FDR = 0.100).

## 4. Discussion

In this study, we examined transcriptomic markers of this substrate switching response in PBMCs following an EBJ intervention, using samples from a previously published short-term clinical study [10]. PBMCs, although primarily immune cells, express a high proportion of the human genome and interact dynamically with metabolic tissues. Through receptors for hormones such as insulin and leptin, PBMCs capture systemic signals that influence metabolic pathways. This positions them as practical “sentinels” for reflecting transcriptional adaptations in key tissues, including adipose, liver, and muscle, which are challenging to biopsy in clinical trials [27]. Prior work shows that PBMCs can mirror fasting-induced changes in lipid metabolism genes observed in adipose tissue and detect diet-induced metabolic shifts, supporting their utility as accessible surrogates for systemic responses in nutrigenomic studies [22,27,28]. At the same time, it is important to recognize their limitations. PBMCs are immune-derived cells, and while they mirror systemic signals, they cannot fully substitute for transcriptional changes occurring in primary metabolic tissues. Thus, while PBMCs provide valuable and biologically coherent insight into systemic nutritional adaptations, their role should be viewed as complementary rather than equivalent to direct tissue profiling. Future work incorporating adipose or muscle biopsies would be required to confirm tissue-level adaptations.

### 4.1. Systemic Absorption and Bioavailability of Anthocyanins from EBJ

In this study, participants consumed American black elderberry juice (*Sambucus nigra* spp. *canadensis*), which contains principally acylated anthocyanins, constituting approximately 70% of the total anthocyanin content when fully ripened, as confirmed in our previous study [9,10]. The prominent acylated anthocyanin is cyanidin-3-(E)-*p*-coumaroyl-sambubioside-5-glucoside, compared to the non-acylated cyanidin 3-O-sambubioside, the predominant form in the European elderberry (*Sambucus nigra*). The latter bears a free hydroxyl group at *C*-5 and lacks acyl functionalities in the structure, as noted by several separate studies investigating the profiles of the European black elderberry [29,30,31,32]. This distinction in anthocyanin composition underscores the critical difference between the two species, as acylation increases stability (to heat, light, and pH) and alters absorption routes relative to non-acylated forms [30].

Acylated anthocyanins are absorbed in the gastrointestinal tract through specific transporters. Computational and in vitro studies demonstrate a preferential interaction with glucose transporter GLUT3 compared to the GLUT1 preference of nonacylated anthocyanins [33,34]. This preferential GLUT3 binding occurs due to structural features of acylated anthocyanins that facilitate binding through both hydrophobic (CH–π and π–π stacking) and hydrophilic (hydrogen bonding) interactions essential for transmembrane transport [34].

Experimentation of GLUT involvement in acylated anthocyanin transport was described by Oliveira et al. [34], who used gold nanoparticles to selectively knockdown GLUT1 and GLUT3 in human gastric epithelial cells. They demonstrated that these transporters account for ~60% of anthocyanin gastric transport, including acylated forms from purple sweet potato (Pn3HBsoph5glc and Pn3HBCsoph5glc), though with lower efficiency than non-acylated counterparts due to steric hindrance. The glucose moiety remained essential for binding regardless of acylation, indicating that acylated anthocyanins like those in American black elderberry can interact with glucose transporters.

Consistent with this purported mechanism, our PBMC transcriptome analysis revealed significant upregulation of *SLC2A3* (GLUT3) at 120 min after EBJ consumption, which may indicate transporter activation extending to systemic circulation beyond gastric interactions. As a high-affinity glucose transporter expressed in leukocytes, this change is consistent with potential modulation of GLUT3 expression in circulating immune cells by EBJ anthocyanins [35]. Overall, the specific anthocyanin profile of American black elderberry and its enrichment in acylated forms is plausibly linked to observed metabolic effects, though direct confirmation in human tissues is needed.

### 4.2. Elderberry Juice and Blood Glucose Homeostasis

In our study, EBJ-fed samples exhibited significant upregulation of key components in insulin signaling and glucose metabolism. This included SLC2A3 (GLUT3) upregulation, which facilitates cellular glucose uptake, RAB10, a Rab GTPase involved in vesicle trafficking for insulin-mediated glucose transport, and PDPK1, a kinase that activates AKT in the PI3K–AKT signaling pathway. In vitro evidence suggests that acylated anthocyanins may induce stronger activation of the PI3K/AKT pathway than their nonacylated counterparts [33].

In the EBJ group, downregulation of PDK2 and PDK4, negative regulators of the pyruvate dehydrogenase complex (PDC), may have facilitated greater glucose utilization. This downregulation facilitates greater entry of pyruvate into the TCA cycle for energy production, allowing for more efficient glucose metabolism following a high-glucose meal. Additionally, we observed upregulation of glycolytic genes, including HK2, PFKFB2, and PFKFB3, suggesting that the glycolytic pathway was also enhanced. These gene expression changes are supported by the 24% reduction in postprandial glucose incremental area under the curve (iAUC) observed in our clinical trial, providing a partial mechanistic basis for the glucose-lowering effects of EBJ [10]. Anthocyanins also reportedly slow intestinal absorption of glucose at the brush border of enterocytes [24]. Therefore, mechanistic events related to intestinal inhibition of glucose absorption by elderberry anthocyanins are also possibly involved but are beyond the scope of the current study.

In contrast, samples from the placebo group showed similar downregulation of PDK4, but this occurred without accompanying reductions in PDK2 or increases in glycolytic genes. Such a pattern may suggest less effective glucose metabolism due to the lack of signaling enhanced by the anthocyanins in the EBJ. Pathway-level analysis indicated that both groups affected glycolysis and gluconeogenesis pathways. However, EBJ uniquely activated compensatory signaling pathways, including insulin signaling, which facilitates glucose uptake, and AMPK signaling, involved in energy switching and fatty acid oxidation. This too is corroborated by the observations on improved insulin sensitivity and reduced respiratory quotient in our previous clinical study.

These findings are consistent with the idea that EBJ may support coordination of glucose-handling pathways and contribute to metabolic flexibility in individuals with overweight or obesity. The differential transcriptomic response suggests that acylated anthocyanins from EJB could influence glucose metabolism, pointing to a potential role in managing metabolic health.

### 4.3. Lipid Metabolism, FAO Suppression, and Mitochondrial Function

In the fed state, insulin drives anabolic lipid metabolism by promoting de novo lipogenesis, a process that balances nutrient storage and metabolic flexibility [36]. Our study revealed distinct metabolic responses in the EBJ group, characterized by a comprehensive modulation of lipid metabolism and mitochondrial function. 

The EBJ group demonstrated significant upregulation of key genes involved in lipid metabolism. ACSL1 and ACSL4 catalyze the activation of long-chain fatty acids for triglyceride synthesis, while ELOVL5 and HACD2/HACD4 facilitate fatty acid chain elongation and lipid diversity. FAR1 supports fatty acid reductase activity, and PPARGC1B is the master regulator of mitochondrial biogenesis and metabolic adaptation. These changes were further supported by coactivators RXRA and NCOA2, which enhance nuclear receptor signaling in lipid metabolism. KEGG pathway analysis confirmed these findings, showing upregulation of glycerophospholipid metabolism and downregulation of fatty acid degradation.

In contrast, the placebo group exhibited only partial gene upregulation, primarily in ACSL4 and FAR1, suggesting a less comprehensive metabolic response.

### 4.4. Fatty Acid Oxidation and Mitochondrial Modulation 

Both groups showed downregulation of FAO genes, including CPT1A and SLC25A20, consistent with insulin’s metabolic effects in the fed state. Notably, the EBJ group exhibited significant downregulation of oxidative phosphorylation, involving critical mitochondrial genes with specific metabolic roles. NDUFV1, a key component of Complex I in the electron transport chain, plays a crucial role in transferring electrons from NADH to ubiquinone, directly impacting energy production. COX3, the final enzyme in the electron transport chain, catalyzes the reduction of molecular oxygen to water, representing the culmination of cellular respiration. The coordinated downregulation of these genes suggests a strategic metabolic adaptation that may prevent nutrient overload, reduce reactive oxygen species (ROS) accumulation, and minimize cellular stress during metabolic adaptation [37]. The above two sections describe a transcriptomic enhancement of lipid synthesis and storage, yet our clinical study observations demonstrated higher fat oxidation over the course of a 3 h postprandial state when study volunteers were fed a high-sugar meal with EBJ compared to a placebo [10]. These transcriptomic and clinical observations may appear contradictory but could potentially be reconciled through the following hypothesis: the dampened insulin response, enhanced glucose handling, and potentially slowed intestinal absorption of the challenge meal’s glucose bolus may help explain why the EBJ treatment led to a comparatively increased whole-body fat oxidation state compared to placebo. At the same time, the transcriptomic analyses suggest the possibility of enhanced switching of metabolic fuels—and thus improved insulin sensitivity—as described below.

### 4.5. Insulin and Nutrient-Sensing Signaling

In both EBJ and placebo groups, transcriptional patterns indicated distinct responses to insulin signaling. In EBJ-fed samples, upregulated genes such as IGF1R, GRB10, and GAB1 indicated enhanced insulin receptor function, supported by GO enrichment for insulin receptor signaling (GO:0008286) and response to insulin (GO:0032868). Activation of the PI3K–AKT pathway was evident through components like PDPK1 and RAB10, along with pathway enrichment linking downstream to mTOR and FoxO signaling. This signaling profile reflects upstream insulin components inhibiting FoxO1 via AKT phosphorylation, thereby promoting anabolic responses [38]. Additionally, an anthocyanin-rich diet enhances insulin sensitivity, as discussed by Randeni et al. [39], who highlighted how anthocyanins modulate the PI3K/AKT pathway, facilitating improved glucose uptake and metabolic responses.

In contrast, placebo-fed samples displayed mixed regulation in insulin-related pathways, including FOXO3, mTOR and FoxO enrichment upregulation, but no significant PI3K–AKT activation. This lack of coordinated anabolic gene expression indicates incomplete adaptation in the placebo group, emphasizing a more robust insulin-driven substrate switching observed in EBJ-fed samples.

### 4.6. Study Limitations

The RNA-seq cohort was modest (n = 10 participants; 40 libraries), which limits external generalizability and precludes adequately powered subgroup or interaction analyses. Nevertheless, the design and analytic framework support internal validity. The 2 × 2 crossover provided four repeated measures per participant, reducing between-person variability and increasing efficiency for treatment contrasts. We modeled repeated measures with lmerSeq, a linear mixed-effects approach specifically developed for small-n RNA-seq designs, which has been shown in simulations and real whole-blood datasets to maintain nominal false discovery rate control and higher power than alternative methods [20]. Broadly, benchmarking studies show that replication increases statistical power more effectively than sequencing depth, with ~6–12 replicates often sufficient depending on design [40]. Similar nutrition-focused crossover transcriptomic studies have successfully reported biologically coherent findings with sample sizes in this same range or smaller (n = 6–14) [41,42,43]. However, our interpretations draw on supportive in vitro, computational, and animal-based research for a mechanistic context, notably with anthocyanin acetylation and transporter interaction. These mechanistic interpretations require direct human evidence. Taken together, our findings should be regarded as exploratory and hypothesis-generating, but are supported by design efficiency, appropriate methods, and field precedent.

## 5. Conclusions

This study provides a comprehensive transcriptomic analysis of American black elderberry juice’s effects on metabolic flexibility in human PBMCs in response to a high sugar test meal. Our findings reveal that elderberry juice (EBJ) consumption may enhance the transcriptional response to feeding, with approximately four times more differentially expressed genes (1512 vs. 350) compared to placebo during the fasted-to-fed transition. This amplified transcriptional plasticity potentially represents a molecular signature of improved metabolic flexibility. The marked enrichment of nutrient-sensing and insulin signaling pathways following EBJ consumption aligns with our previous clinical findings showing improved glucose tolerance and higher fat oxidation compared to placebo [10]. Our GSEA results demonstrate that EBJ upregulated key nutrient-sensing pathways (FoxO, insulin, mTOR, AMPK, cAMP/cGMP-PKG) and autophagy, while simultaneously affecting energy metabolism pathways (oxidative phosphorylation, TCA cycle, glycolysis). This pattern suggests that EBJ enhances cellular energy efficiency by promoting dynamic fuel switching and optimizing metabolic resource allocation instead of increasing overall metabolic rate, which was not observed in our clinical findings. The transcriptional changes in key metabolic genes provide mechanistic insights into EBJ’s physiological effects. Particularly notable was the stronger downregulation of PDK4 (pyruvate dehydrogenase kinase 4) in EBJ-fed samples compared to placebo, indicating enhanced suppression of PDK4’s inhibitory effect on carbohydrate oxidation. Paired with a reduction in genes responsible for electron transport chain enzymes, this could suggest an enhancement in replenishing TCA cycle intermediates, or protection of the mitochondria against ROS, but molecular investigations would need to confirm this. These insights stand in juxtaposition with the increased fat oxidation observed in our previous study [10], where EBJ consumption significantly lowered the respiratory quotient and, therefore, increased fat utilization during both postprandial and exercise conditions; however, a dampened insulin response in the EBJ treatment hypothetically explains this disparity between transcriptomics and clinical observations. The upregulation of PPARGC1B and AMPK, key regulators of mitochondrial biogenesis and fatty acid oxidation, shows that EBJ enhances overall oxidative capacity, further supporting metabolic flexibility. Our findings are consistent with recent research on elderberry anthocyanins’ bioavailability and metabolic effects. The rapid absorption of anthocyanins in the stomach via transporters like GLUTs and bilitranslocase [44] potentially explains how elderberry compounds can exert acute effects on gene expression in peripheral tissues. Notably, our finding that SLC2A3 (GLUT3) was upregulated in PBMCs after EBJ intervention aligns with our previous discussion [24], where we identified GLUT3 as a major transporter involved in anthocyanin absorption in gastric cells, and the present study extends its potential modulation to white blood cells. These converging results suggest that anthocyanin-rich foods may acutely shape glucose transporter expression, potentially supporting both anthocyanin bioavailability and glucose metabolism at multiple biological sites. These results support exploring EBJ as a practical dietary adjunct for enhancing metabolic flexibility—including improved glucose handling, insulin sensitivity, and fat oxidation—in overweight individuals, potentially reducing reliance on pharmacological options for managing metabolic health. Nevertheless, given the modest RNA-seq cohort (n = 10), these transcriptomic findings should be considered exploratory and hypothesis-generating, with confirmation warranted in larger, adequately powered studies.

## Figures and Tables

**Table 1:** Fisher’s exact test of pathway enrichment during the fasted-to-fed transition. Counts of significantly differentially expressed genes (DEGs; FDR-adjusted *p* < 0.05, |log_2_FC| > 0.25) for the elderberry juice (EBJ) and placebo (PL) groups, and those shared between groups. Pathways were selected from a whitelist of KEGG metabolic pathways and additional literature-curated gene sets. FDR (false discovery rate) denotes the expected proportion of false positives after multiple-testing correction. Data reflect pooled transcriptomic analyses from n = 10 participants per group.

Pathway	EBJ Count	PL Count	Shared Count	FDR
Insulin Signaling	18	7	6	1.56E-05
FoxO Signaling	8	3	3	<0.001
Literature Curation	4	2	2	0.001
MAPK Signaling	21	8	5	0.001
Circadian Rhythm	4	2	2	0.001
PI3K–Akt Signaling	29	9	5	0.009
Autophagy	17	5	3	0.013
Cytokine–Cytokine Receptor Interaction	19	5	3	0.015

## Data Availability

Research data from measures of human transcriptomics are available in Supplementary Tables S1–S7 described above.

## Supplementary Materials

The following supporting information can be downloaded at: https://www.mdpi.com/article/10.3390/nu17193142/s1, Table S1: Custom metabolic flexibility gene whitelist constructed from 38 KEGG pathways related to energy metabolism, nutrient sensing, redox regulation, and mitochondrial function; Table S2: Full transcriptomic analysis results for all genes; Table S3: Metabolic flexibility–related differentially expressed genes (DEGs) identified during the fasted-to-fed transition in EBJ and PL groups; Table S4: Fisher’s exact test results for differential pathway representation between treatment arms; Table S5: Gene Ontology enrichment results for DEGs in each treatment group; Table S6: KEGG gene set enrichment analysis (GSEA) results for all pathways in EBJ and PL groups; Table S7: Filtered KEGG GSEA results for curated metabolic flexibility–related pathways (FDR < 0.1).

## Abbreviations

The following abbreviations are used in this manuscript:

AMPK	AMP-activated protein kinase
BP	Biological Process (Gene Ontology category)
CC	Cellular Component (Gene Ontology category)
C3GE	Cyanidin-3-glucoside equivalents
DEG	Differentially expressed gene
EBJ	Elderberry juice
FAO	Fatty acid oxidation
FC	Fold change
FDR	False discovery rate
FoxO	Forkhead box O
GSEA	Gene set enrichment analysis
GO	Gene Ontology
HK2	Hexokinase 2
KEGG	Kyoto Encyclopedia of Genes and Genomes
MAPK	Mitogen-activated protein kinase
MF	Molecular Function (Gene Ontology category)
mTOR	Mechanistic target of rapamycin
MTT	Meal tolerance test
NES	Normalized enrichment score
PBMC	Peripheral blood mononuclear cell
PDK2	Pyruvate dehydrogenase kinase 2
PDK4	Pyruvate dehydrogenase kinase 4
PI3K–AKT	Phosphoinositide 3-kinase–protein kinase B
PL	Placebo
PPARGC1B	Peroxisome proliferator-activated receptor gamma coactivator 1-beta
RQ	Respiratory quotient
TCA	Tricarboxylic acid
TOR	Target of rapamycin
VST	Variance-stabilizing transformation
